# Global overview of short-term alcohol policies: need for global exchange of experience and impact measurement

**DOI:** 10.1192/bji.2025.16

**Published:** 2026-08

**Authors:** Basma Al-Ansari, Venkata Lakshmi Narasimha, Beatrice L. Matanje, Enjeline Hanafi, Francina Fonseca, Jenna L. Butner, Jiang Long, Djibril Moussa, Laura Orsolini, Mehdi Farokhnia, Katrine Melby, Preethy Kathiresan, Sung Young Huh, Roshan Bhad, Christos Kouimtsidis, Alex Baldacchino

**Affiliations:** 1 Academic, Menzies Centre for Health Policy and Economics, Sydney School of Public Health, Faculty of Medicine and Health, The University of Sydney, Sydney, New South Wales, Australia; 2 Assistant Professor, Centre for Addiction Medicine, Department of Psychiatry, National Institute of Mental Health and Neurosciences (NIMHANS), Bengaluru, India.; 3 Chief Executive Officer, National AIDS Commission, Lilongwe, Malawi; 4 Medical Staff, Department of Psychiatry, Faculty of Medicine Universitas Indonesia, Dr Cipto Mangunkusumo General Hospital, Jakarta, Indonesia; 5 Head of Addictions Section, Mental Health Institute and Research Institute, Hospital del Mar, Barcelona, Spain; 6 Assistant Professor Adjunct, Department of Medicine, Yale University School of Medicine, New Haven, Connecticut, USA; 7 Psychiatrist, Shanghai Mental Health Center, Shanghai Jiao Tong University School of Medicine, Shanghai, China; 8 Atlantic Fellow, Global Brain Health Institute, UCSF, San Francisco, California, USA; 9 Assistant Professor, Unit of Clinical Psychiatry, Department of Neurosciences/DIMSC, Polytechnic University of Marche, Ancona, Italy; 10 Staff Scientist, National Institute on Drug Abuse and National Institute on Alcohol Abuse and Alcoholism, National Institutes of Health Intramural Research Program, Baltimore, Maryland, USA; 11 Specialist Physician, Clinic of Substance Use and Addiction Medicine, St Olav University Hospital, Trondheim, Norway; 12 Assistant Professor, Department of Psychiatry, All India Institute of Medical Sciences (AIIMS), New Delhi, India; 13 Assistant Professor, Department of Psychiatry, Pusan National University Yangsan Hospital, Yangsan, South Korea; 14 Additional Professor of Psychiatry at National Drug Dependence Treatment Centre (NDDTC), All India Institute of Medical Sciences (AIIMS), New Delhi, India; 15 Consultant Psychiatrist, Medical School, Imperial College London, Surrey and Borders Partnership NHS Foundation Trust, London, UK; 16 Professor of Medicine, Medicine, Psychiatry and Addictions, School of Medicine, University of St Andrews, St Andrews, UK

**Keywords:** Alcohol, alcohol sale, ban, policy, public health, short-term restriction

## Abstract

Alcohol use disorder is a global public health concern and national policies are often implemented to help control alcohol consumption and related consequences. Increasingly, many countries are resorting to transient (short-term) alcohol policies which are implemented for a restricted period of time as an action plan for particular events or health-related issues. The COVID-19 pandemic emphasised the need for rapid decision-making and short-term fast-acting policies. This paper discusses contexts in which these transient policies are used and highlights the need for impact measurement and global exchange of experiences. This is particularly important to avoid gaps that the global alcohol industry could utilise to expand its influence and market.

Alcohol consumption is one of the leading risk factors for death and disability worldwide.^1^ Many policies have been introduced across the world to reduce alcohol-related harm.^2^ These policies are mainly implemented as part of national alcohol plans for particular countries.^1^ However, some types of alcohol policy, identified here as short-term policies, are implemented occasionally as a strategic response to a temporary challenge or a particular situation for a specific period. These policies have not necessarily been put into action by most countries. This paper aims to discuss contexts in which these short-term policies are applied, using examples from countries in which members of the International Society of Addiction Medicine’s New Professionals Exploration, Training & Education Committee (ISAM-NExT) have experience. The paper then highlights the need for impact measurement and empirical evaluation of the public health effects of such policies. It also calls for a global exchange of experiences to better understand and address the challenges such as those posed by the alcohol industry.

## Method

In addition to fixed national alcohol policies, some countries implement short-term policies – these are temporary measures or regulations enacted by the national or local government to manage or control the consumption, sale and distribution of alcoholic beverages for a limited period. They are made to address specific issues or events, such as public health crises (as in the COVID-19 pandemic), holidays or mass gatherings, where alcohol might play a significant role in causing public disorder. Short-term alcohol policies may act on top of existing regulations.

This definition of short-term policies was shared with ISAM-NExT Committee members, and they were asked whether such policies are implemented in their countries of expertise and for what reason. After 1 month, responses were received from 18 countries, ten of which were included in our study (USA, Norway, South Korea, India, Qatar, Japan, China, Australia, Turkey and Indonesia; Table 1). Members from four countries had no experience with such policies, while four reported on non-applicable ones. The ISAM-Next Committee is a global platform for early-career addiction medicine professionals that currently has 30 early-career addiction professional members from 22 countries.

**Table 1:** A snapshot of the contexts in which short-term alcohol policies are implemented Table 1 long description.

Context	Event/reason	Alcohol policy response	Implications	Country	Duration
**Pandemic response (COVID-19 pandemic)**	To limit the spread of COVID-19	Social distancing plan	Decrease in alcohol consumption among adolescents	South Korea^8^	2 years (2020 and 2021)
	State alcohol-related laws during the COVID-19 to mitigate its spread	Capacity restrictions for bars and restaurants Reduced operating hours Mandates to serve alcohol outdoors only Introducing online alcohol purchase and home delivery	Online sale of alcohol led to increase in alcohol-related harm	USA^6^	2 years (2020 and 2021)
	To combat the COVID-19 pandemic	Restrictions on social events, travelling and on-premises trading hours Closing the borders	56.8% of respondents reported no change in drinking, 29.9% reported less drinking and 13.3% reported more drinking, with little overall change in alcohol consumption 23% increase in domestic sales during the pandemic period	Norway^7,20^	Restrictions: 2 years (2020 and 2021) Border closure: March–December 2020
		‘Corona tax’ (70% excise duty on alcohol) during COVID-19 pandemic in Delhi and complete ban on sale of alcohol during the initial lockdown	Reduction in short-term demand by 30–90% peak sales Stockpiling of alcohol Buying alcohol from neighbouring states (if restricted to few states only)	India^3^	Several weeks
**Global mass gatherings**	FIFA World Cup 2022	Selling alcoholic beverages at FIFA Fan Festival, other fan destinations and licensed venues, but sales points of beer removed from the perimeter of the stadium	Impact on economy, tourism and entertainment industry needs evaluation	Qatar^11^	During FIFA event
	Tokyo Olympics 2021	Ban on sale of alcohol at the venue	Data unavailable to assess the impact	Japan^9^	During the event
**To reduce alcohol-related harms**	Increase in crime rate	Limits on alcohol sales in Alice Springs, Australia	Reduction in domestic violence and alcohol-related emergency department visits	Australia^12^	3 months then extended to 3 years
	Increase in alcohol-related road traffic accidents during New Year	Nationwide crackdown on drink-driving	Data unavailable to assess the impact	South Korea^23^	2–3 months
	To reduce harmful driving under the influence of alcohol during summer	Nationwide crackdown policy and arrest	Criminal laws are proven to be effective in reducing the harm associated with alcohol, but no data are available on this short-term policy	China^13^	2 days
**Alcohol bans for specific occasions**	Holy month of Ramadan	Alcohol ban	Data unavailable to assess the impact but there are fewer admissions to emergency departments because of lifestyle change	Turkey^15^ and other Muslim majority countries with restricted alcohol availability, e.g. Egypt/Malaysia^10^	1 month
	National/state elections	Ban on alcohol sale	Increase in alcohol-related complications among dependent users due to sudden ban/withdrawal	India^14^	Every 5 years (3 days before election and during counting of votes)

We used thematic analysis to analyse the responses, identifying common contexts with no presumptions.

## Results

Four contextual reasons were identified for implementing short-term policies: unplanned in response to unexpected events (e.g. pandemic response); planned in response to expected short-term events (e.g. global mass gatherings); planned to mitigate harm (e.g. reducing alcohol-related harms); and planned in response to longer-term systemic events (e.g. alcohol bans during specific occasions).

### Pandemic response

In response to the COVID-19 pandemic, many short-term alcohol policies were implemented around the world^3,4^ in line with World Health Organization’s recommendation of limiting drinking to one drink per day during the pandemic. Despite some studies^3^ the impact needs to be further explored.

Certain countries prohibited the sale of alcohol during lockdown periods, whereas others classified it as a necessity, leading to various global challenges.^3^ For example, Australia temporarily relaxed liquor licensing, allowing restaurants, cafes and small bars to sell alcohol for takeaway and delivery. As a result, in New South Wales, Australia, alcohol consumption increased, resulting in increased psychological stress, domestic violence and child abuse.^5^ Similarly, USA home delivery led to increased alcohol-related harm.^6^

In Norway, for instance, little change in alcohol consumption was observed in the 3 first months after extending the provisions of the Infection Control Act in March 2020, with restrictions on social events, travel and on-premises trading hours.^7^ Over half of responders in a study from June to July 2020 reported no change in drinking, one-third reported less drinking and only 13% reported increased consumption. Those with a low past-year consumption drank less, whereas those with a initially high consumption drank more.^7^ On the other hand, in South Korea, social distancing and school closures during the pandemic notably reduced consumption among adolescents owing to decreased peer pressure.^8^

### Global mass gatherings

Linking both the COVID-19 pandemic and global mass gatherings, some countries had to apply short-term policies to enable them to host such gatherings during the pandemic. Despite the norm, alcohol-containing beverages were forbidden in the Olympic villages during the Tokyo Olympics in 2021.^9^ There are no available data to assess the impact of this particular tactic. However, in countries in which alcohol is typically banned or highly restricted, such as Muslim majority countries (MMCs),^10^ short-term policies are often applied to the availability and consumption of alcohol. For example, Qatar implemented a short-term policy on easing the restrictions of alcohol availability and consumption when it hosted football’s FIFA World Cup in 2022.^11^ The impact is not clear, as many of the fans came from neighbouring MMCs with a culture of limited exposure to alcohol consumption.

### To reduce alcohol-related harms

In some countries, short-term alcohol restrictions are implemented as a response to increased alcohol-related crime in a specific region. Australia, for instance, implemented alcohol restrictions in Alice Springs for an initial period of 3 months, then extended to 3 years. This approach resulted in a reduction in crime but it is not yet considered an evidence-based intervention.^12^

Alcohol-related motor vehicle accidents and injuries have a global impact and so enforcing criminal laws effectively reduces these harms. In China, a 2-day nationwide crackdown on drink-driving resulted in the arrest of 17 000 individuals for driving under the influence of alcohol.^13^ It is vital to investigate how effective this sharp 2-day policy has been for it to be considered for implementation in other countries.

### Alcohol bans for specific occasions

Transient policies are also used on occasions where alcohol sales and/or consumption is banned for a defined period. An example of this is the ban on alcohol sales in India that starts 3 days before national and state-level elections (once every 5 years) and continues during the vote tallying period.^14^ Although the socioeconomic impact of election-period bans has not been studied, the evidence for harm in dependent users accumulates.^14^ There are increased cases of severe alcohol withdrawal syndrome and presentations to emergency departments,^14^ as well as an increase in illicit liquor sales. The risk of consumption of illegally produced alcohol and associated health hazards is also elevated.^14^

On the other hand, the adverse effects are not the same when a transient ban is implemented in an MMC population during a religious period such as Ramadan.^15^ Limited data show evidence of decreased admissions to emergency departments.^15^ This can be explained by the pre-existing religious restrictions and stigma surrounding alcohol availability and consumption in this context.^10^

## Discussion

### Short-term policies and the global alcohol industry

The global alcohol industry is known for hunting opportunities to influence alcohol policy-making in favour of its own interests rather than public health.^16^ In the contexts discussed above, there are some gaps that the alcohol industry might use to strengthen its presence.

For example, the industry challenged^17^ the implementation of short-term policies during COVID-19, as its strong online and social media presence both encouraged people to buy alcohol online and benefitted the industry owing to limited regulation of online sales.^18^

Mass gatherings such as the FIFA World Cup and the Olympic Games often become targets for the alcohol industry, which sponsors events and shapes alcohol policies, from availability and advertising to serving intoxicated individuals.^19^ This influence challenges host countries’ ability to enforce short-term alcohol policies effectively. For instance, despite the UK’s extensive experience, the industry’s tactics to circumvent restrictions on alcohol advertising have highlighted the country’s lack of self-regulation in marketing.^19^ Addressing these issues is crucial for better managing the industry’s impact on alcohol policies during such significant events.

Although no information is available regarding the effects of the alcohol ban during the Tokyo Olympics^9^ in 2021 and Beijing Winter Olympics in 2022, investigating approaches like this may provide new ways to address the influence of the alcohol industry during global events.

Qatar was the first MMC to host the FIFA World Cup as a global event. This presented challenges both for the alcohol industry, which has a strong reputation for sponsoring the World Cup, and for Qatar as a country with alcohol restriction/prohibition.^10^ Highlighting the challenges posed to existing policies on alcohol in Qatar and how the country adapted to host this global event is crucial.^11^ This is particularly relevant as FIFA looks to expand its events into Islamic countries such as Saudi Arabia. Moreover, Saudi Arabia, Egypt and Greece are contemplating a collaborative proposal for hosting the 2030 men’s World Cup.^11^

European countries tend to have stricter regulations and policies on the import of alcohol compared with MMCs. During the COVID-19 pandemic, increasing domestic sales could be seen after border closures in Norway.^20^ In addition to weak or absent alcohol policies, many Middle Eastern countries, plagued by war and government instability, have weak or non-existent border protections or taxes.^10,21^ This creates opportunities for the global alcohol industry to use short-term policies to exploit these ‘dry’ regions as new markets, distributing alcohol legally or illegally.

A further concern that needs to be addressed is whether some of these policies might be made by the alcohol industry itself. For example, the Korean Alcohol Research Foundation was established by the alcohol industry in 2000.^22^ One notable initiative is the ongoing Stop Drink Driving Campaign, which began in 2002 and utilises booklets, posters and advertisements to raise awareness.^22^ Sometimes raising awareness can include the national short-term crackdown policies on drink-driving.^23^

### Need for improved impact measurement

To aid policymakers and service providers in crafting effective short-term alcohol policies, research on their impacts must be current, consistent and uniform. It is essential to balance the targeted and unintended outcomes of these policies by investing in treatment services and managing supply and demand reduction efforts.^12^ For example, a policy might achieve the targeted outcome that it was designed for, such as crime reduction in Alice Springs, Australia,^12^ but it might also have unwanted health outcomes, such as the severe complications in alcohol-dependent individuals observed during election-period bans on alcohol sales in India.^14^ Balancing the positive and negative impacts of policies needs careful planning and consideration of the context in which they are implemented. There has been some reported evidence on outcomes at a national level in some countries,^12^ but this is considered to be soft data. Therefore, it is essential to build on existing experience by studying the impact of short-term alcohol policies precisely and exchanging those details between the global community. This is particularly important because the World Health Organization’s global strategy to reduce the harmful use of alcohol emphasises national alcohol policies with a long-term focus.^2^ Short-term policies have not been explicitly included in those indicators or recommendations.

Countries’ differing policies lead to varied experiences. Measuring their impact and sharing insights can enhance policy effectiveness and understanding. For instance, studies might track health outcomes such as alcohol-related hospital visits, injuries, poisonings and mortality rates, or assess social and behavioural effects by analysing changes in alcohol-related crimes, drink-driving incidents and public disorder.

In conclusion, short-term alcohol policies complement national strategies for specific situations and durations. These policies are applied in contexts such as pandemic response or global mass gatherings and vary widely in their outcomes. The COVID-19 pandemic, for example, emphasised the need for rapid decision-making and short-term fast-acting policies.

There is a need to achieve an equilibrium between the targeted and unwanted outcomes of these policies. This can lead into an evidence-based response where a balance is made between investing in treatment and services available and the supply and demand reduction. Therefore, drawing parallels between similar contextual factors globally where short-term alcohol policies are implemented can help in advancing the process of preparedness for a safe-for-all implementation and so investing in the benefits that these policies are designed for.

Questioning the alcohol industry’s influence and benefits highlights the importance of focusing on impact measurement and global knowledge exchange. This is vital for countries with limited policies on alcohol, such as those that ban alcohol consumption on religious grounds.

Establishing a global database for short-term alcohol policies and standardising evaluation metrics can support international collaboration, enhance data sharing, and strengthen policy adaptation and knowledge exchange.

## Data Availability

The authors confirm that the data supporting the findings of this study are available within the article.

## Table 1 Long description

The table has six columns: Context, Event/reason, Alcohol policy response, Implications, Country, and Duration. It contains data on short-term alcohol policies implemented in various countries. Row 1: Pandemic response (COVID-19 pandemic), To limit the spread of COVID-19, Social distancing plan, Decrease in alcohol consumption among adolescents, South Korea, 2 years (2020 and 2021). Row 2: Pandemic response (COVID-19 pandemic), State alcohol-related laws during the COVID-19 to mitigate its spread, Capacity restrictions for bars and restaurants, Online sale of alcohol led to increase in alcohol-related harm, USA, 2 years (2020 and 2021). Row 3: Pandemic response (COVID-19 pandemic), To combat the COVID-19 pandemic, Restrictions on social events, 56.8 percent of respondents reported no change in drinking, Norway, Restrictions: 2 years (2020 and 2021), Border closure: March-December 2020. Row 4: Pandemic response (COVID-19 pandemic), To combat the COVID-19 pandemic, Corona tax (70 percent excise duty on alcohol) during COVID-19 pandemic in Delhi and complete ban on sale of alcohol during the initial lockdown, Reduction in short-term demand by 30-90 percent peak sales, India, Several weeks. Row 5: Global mass gatherings, FIFA World Cup 2022, Selling alcoholic beverages at FIFA Fan Festival, other fan destinations and licensed venues, but sales points of beer removed from the perimeter of the stadium, Impact on economy, tourism and entertainment industry needs evaluation, Qatar, During FIFA event. Row 6: Global mass gatherings, Tokyo Olympics 2021, Ban on sale of alcohol at the venue, Data unavailable to assess the impact, Japan, During the event. Row 7: To reduce alcohol-related harms, Increase in crime rate, Limits on alcohol sales in Alice Springs, Australia, Reduction in domestic violence and alcohol-related emergency department visits, Australia, 3 months then extended to 3 years. Row 8: To reduce alcohol-related harms, Increase in alcohol-related road traffic accidents during New Year, Nationwide crackdown on drink-driving, Data unavailable to assess the impact, South Korea, 2-3 months. Row 9: To reduce harmful driving under the influence of alcohol during summer, Nationwide crackdown policy and arrest, Criminal laws are proven to be effective in reducing the harm associated with alcohol, but no data are available on this short-term policy, China, 2 days. Row 10: Alcohol bans for specific occasions, Holy month of Ramadan, Alcohol ban, Data unavailable to assess the impact but there are fewer admissions to emergency departments because of lifestyle change, Turkey and other Muslim majority countries with restricted alcohol availability, e.g. Egypt/Malaysia, 1 month. Row 11: Alcohol bans for specific occasions, National/state elections, Ban on alcohol sale, Increase in alcohol-related complications among dependent users due to sudden ban/withdrawal, India, Every 5 years (3 days before election and during counting of votes).

Navigate back to Table 1.
